# “In our community, we normalize pain”: discussions around menstruation and uterine fibroids with Black women and Latinas

**DOI:** 10.1186/s12905-024-03008-z

**Published:** 2024-04-12

**Authors:** Minerva Orellana, Karen. N DSouza, Jane Q. Yap, Abhirami Sriganeshan, M. Elena Jones, Charis Johnson, Megan Allyse, Sateria Venable, Elizabeth A. Stewart, Felicity Enders, Joyce E. Balls-Berry

**Affiliations:** 1 Department of Obstetrics and Gynecology, University of Washington, Seattle, WA USA; 2 Center for Clinical and Translational Science, Mayo Clinic, Rochester, MN USA; 3 Department of Quantitative Health Sciences Research, Mayo Clinic, Rochester, MN USA; 4 University of Miami, Miami, FL USA; 5 Department of Neurology, Washington University School of Medicine, 4488 Forest Park, St. Louis, MO 63108 USA; 6 Program in Biomedical Ethics Research, Mayo Clinic, Rochester, MN USA; 7 Department of Obstetrics and Gynecology, Mayo Clinic, Rochester, MN USA; 8 The Fibroid Foundation, Bethesda, MD USA; 9 Division of Reproductive Endocrinology, Department of Obstetrics & Gynecology, Mayo Clinic, Rochester, MN USA; 10 Mayo Clinic College of Medicine & Science, Rochester, MN USA; 11 Division of Clinical Trials and Biostatistics, Mayo Clinic, Rochester, MN USA

**Keywords:** Health disparities, Uterine fibroids, Women’s health, Community-engaged research, Leiomyomas, Menstruation, Taboo, Latinas, Black women

## Abstract

**Background:**

Uterine fibroids are non-cancerous neoplasms that arise from the uterus affecting over 75% of women. However, there is a disparity with Black women having an increased prevalence of nearly 80%. Black women also experience increased symptom burden, including younger age at the time of diagnosis and increased number and volume of fibroids. Less is known about other ethnoracially diverse women such as Latinas and the potential cultural impacts on fibroid burden and treatment.

**Methods:**

Community engagement studios were conducted to facilitate discussions with stakeholders on their uterine fibroid and menstruation experience. We recruited Black women (*n* = 6) diagnosed with uterine fibroids and Latinas (*n* = 7) without uterine fibroids. We held two virtual community engagement studios split by uterine fibroid diagnosis. The studios were not audio recorded and notes were taken by four notetakers. The notes were thematically analyzed in Atlas.ti using content analysis.

**Results:**

Participants felt there was a lack of discussion around menstruation overall, whether in the home or school settings. This lack of menstruation education was pronounced when participants had their first menstruation experience, with many unaware of what to expect. This silence around menstruation led to a normalization of painful menstruation symptoms. When it came to different treatment options for uterine fibroids, some women wanted to explore alternative treatments but were dismissed by their healthcare providers. Many participants advocated for having discussions with their healthcare provider about life goals to discuss different treatment options for their uterine fibroids.

**Conclusion:**

Despite uterine fibroid diagnosis, there is silence around menstruation. Menstruation is a normal biological occurrence and needs to be discussed to help prevent delayed diagnosis of uterine fibroids and possibly other gynecological disorders. Along with increased discussions around menstruation, further discussion is needed between healthcare providers and uterine fibroid patients to explore appropriate treatment options.

## Background

Uterine fibroids (UF), also known as leiomyomas, are non-cancerous neoplasms that have a lifetime prevalence in > 75% of women [1]. Prevalence is often thought to be underestimated due to high rates of asymptomatic women, as well as limitations in study population and diagnostic methodology [2]. Up to 50% of UF patients are identified as symptomatic and experience a variety of symptoms with differing severity, including heavy and/or prolonged menstrual bleeding, anemia, increased urinary frequency, pelvic pain/pressure, bowel dysfunction, and abdominal protrusion [2, 3]. UF are a known contributor to reproductive dysfunction, including subfertility, infertility, and miscarriage; UF are observed in 5–10% of infertility patients, and 27% of patients seeking assisted reproductive fertility care [4].

Risk factors for UF diagnosis include age up to menopause, familial history, premenopausal state, hypertension, and diet including a vitamin D deficiency [2, 5, 6]. Non-Hispanic Black/African American (NHB) women have a higher UF prevalence, experience UF at an earlier age, and experience a higher disease burden compared to non-Hispanic White women (NHW) [2, 3, 7–10]. These disparities are observed in the treatment and health outcomes of UF. NHB women are almost seven times as likely to undergo a myomectomy and more than two times as likely to undergo a hysterectomy [11]. At the time of hysterectomy, NHB women are often premenopausal, and experience quality-of-life-impairing, severe, symptomology [9, 12]. In contrast to NHW women, NHB women are four times as likely to develop post-operative complications and are nearly three times as likely to be hospitalized, with a higher likelihood of mortality [9, 13]. Less is known about other ethnoracial minority groups, especially Hispanic/Latina (HL) women, which represent the fastest-growing population in the United States (US) [2, 3, 14, 15].

Early diagnosis is essential to improving the quality of life and reproductive health of UF patients, yet a relative lack of awareness of UF – by both patients and providers - and its symptoms delays diagnosis; a 2013 survey reported it took patients an average of 3.6 years to seek treatment, and 41% saw at least two healthcare providers prior to diagnosis [2, 3, 12, 16]. Some of this delay can be attributed to the perception that providers dismiss symptoms as normal menstruation or recommending oral contraceptives as a “quick fix” [17].

Findings from our group’s 2021 study suggest that there are sociocultural factors that cause this lack of awareness and lead to diagnostic and treatment delays [3]. These same sociocultural factors impact the recruitment of ethnoracially diverse women in research studies. Recent work has begun to investigate how ethnoracially diverse women, especially HL women, learn about research studies and identify their motivators for participating in such studies [15].

To understand the limited awareness regarding UF and sociocultural factors, we conducted a series of community engagement studios (CES) with NHB and HL community stakeholders to identify topics around UF and menstruation and the impact of sociocultural influences. The topics will then be implemented in our future work to create an interview guide for our national study recruiting NHB and HL with UF. This manuscript will focus on the preliminary topics and experiences from NHB and HL women.

## Methods

### Research team

The research team consisted of ethnoracially diverse women researchers across two institutions, Mayo Clinic and Washington University in St. Louis. The team has been trained in both community-engaged and qualitative research methodologies.

### Participant recruitment

Community members were eligible to participate if they were biologically women and had an ethnoracial self-identification of NHB or HL. Convenience and feasibility sampling methods were used to recruit interested community members via thethe researcher team’s personal networks and social media. Two groups of women were recruited, (1) NHB women with a UF diagnosis, since NHB women have a higher lifetime prevalence of UF, and (2) HL women without a UF diagnosis. UF diagnosis was self-reported by participants. We were interested in discussions around menstrual experiences in women with and without UF. For the HL group, we were interested in determining cultural practices around menstruation as it is still unclear if they are at an increased risk for UF. For NHB, we asked additional questions on UF due to UF disparity. Recruited women were asked to participate in one of two community engagement studios.

### Community engagement studios

A community engagement studio (CES) is a structured method for obtaining input from stakeholders to enhance the design, conduct, and dissemination of research [18]. CES were chosen to provide insight into the best practices for creating and implementing culturally appropriate studies with ethnoracially diverse women regarding topics of menstruation and UF. CES consist of 5–8 stakeholders participating in a virtual facilitated group discussion led by a trained qualitative researcher. The lead researcher (MO) shared a brief presentation on the research topic and goals of the CES. Both CES had an ethnoracially congruent facilitator (JBB and MEJ). The CES were completely confidential and were not video or audio recorded, but detailed notes were taken by trained researchers (MO, JQY, AS, CJ).

### Theoretical framework

The interview guide was developed using prior research to expand on cultural influences on UF treatment management along with the Health Belief Model (HBM) [3]. HBM is a behavioral psychological model which posits that a person’s beliefs can predict their health behavior [19]. While this model consists of six constructs, we only utilized two: perceived barriers and perceived benefits, which are the most powerful of the HBM construct [20]. Perceived barriers convey that taking action would outweigh the benefits. Perceived benefits refer to a scenario where benefits outweigh taking action.

### Data analysis

We utilized content analysis to analyze the CES notes using Atlas.ti software. Content analysis was used to create themes based on similar meanings and allowed both inductive and deductive (*a priori* themes) coding [21–23].

There was a total of 27 pages of notes (*n* = 15 HL, *n* = 12 NHB) from four trained researchers (MO, JQY, AS, CJ). Two different codebooks were created based on UF diagnosis. To create the codebook, three coders (MO, JQY, AS) initially analyzed four pages (23%) and four pages (26.7%) for the HL and NHB CES, respectively. The rest of the notes were coded through consensus coding.

This paper will report on the selected topics that arose from the CES from our NBH and HL stakeholders.

## Results

### Demographics

We held two CES, the first with six NHB biological females who were diagnosed with UF, and the second with seven HL biological females without a UF diagnosis. Both CES were conducted in English. However, some HL participants responded in Spanish which was facilitated by our bilingual HL facilitator. We were only able to obtain the demographic data for five HL participants due to lack of completion of post-CES survey. The average age of NHB participants was 47.5 years and HL was 39.2 years. A majority of the participants were fully employed (81.8%). Demographics are listed in Table 1.

**Table 1:** Demographics of Community Engagement Studio Participants. There is only demographics for five of the seven Hispanic/Latinas participants

Demographics	Black/African American participants (*N* = 6)	Hispanic/Latina participants (*N* = 5 of 7)
Biological female	6 (100%)	5 (71%)
Female gender identity	6 (100%)	5 (71%)
**Employment status**		
Full time	5 (83.3%)	4 (57%)
Unemployed	1 (16.7%)	1 (14%)
**Age (in years)**		
Mean (SD)	47.5 (5.5)	39.2 (9.9)

### Menstruation discussions

Participants overwhelmingly reported a lack of discussion around menstruation, both in K-12 educational settings and at home. However, we asked participants to provide insight into the best way to start a conversation with women about their menstrual experiences for our future study.

“Ask details about their cycle.” – Black woman participant

“How do you feel when you go to the physician? Are you telling him [them] everything?” - Black woman participant

One participant described how uncomfortable she felt talking to healthcare practitioners due to the presence of her mother at the appointments growing up so she couldn’t talk about menstruation with her provider.

“I couldn’t talk directly [to the doctor] because my mom was there and couldn’t be comfortable.” – Latina participant

Some of the participant’s parents expected the school system to teach their children about menstruation, yet were still uncomfortable since it was combined with sexual education. This resulted in many participants not having adequate information about menstruation and its symptoms.

“My parents relied on information from school, but I was in a Catholic school…. I didn’t know what was normal [menstruation symptoms].”

– Latina participant

“Sex and menstruation go hand in hand, so both are taboo.” - Latina participant

### Menarche experiences

For their first menstrual experience, many participants were unaware of what was to be expected during their menstrual cycles, as they were not informed by family members or the school system.

“Nobody…told me about it [menstruation] – Latina participant

“Personally, [I] got it really young and [my] mom didn’t see it [my period] coming.” - Latina participant

### Normalization of menstrual symptoms

Multiple participants from both CES talked about the silence surrounding menstruation experiences and painful symptoms. This is connected to how Black and Brown women are expected to withstand pain and stay silent. Much of the discussion centered around cultural norms and navigating menstruation.

“Cultures are not good at talking about menstruation because of this sex taboo.” – Latina participant

“Even today… there is a lot of shame in our own body and our own blood… and that is likely cultural.” – Latina participant

“People want to have a cloak of silence to not appear weak.” – Black woman participant

“In the Black and Hispanic communities, we have this thing that we are strong and can just endure what is happening…We have to get over the facade that we need to endure everything just because periods are a normal part of our bodies.” – Black woman participant

### Uterine fibroid symptomology, diagnosis, and treatment

In some women, abnormal symptoms, such as heavy periods, were normalized and delayed their UF diagnosis. One participant experienced extremely painful symptoms that resulted in her going to the emergency room where she was diagnosed with UF.

“Black women think that it’s normal or acceptable to have heavy or painful periods.” – Black woman participant

“I was diagnosed in the ER [with uterine fibroids].” – Black woman participant

“Our body isn’t meant to withstand pain. It’s not good.” – Black woman participant

### Alternate uterine fibroid treatment discussions

In the NHB CES, some women mentioned seeking alternative treatments for their UF and that their physicians were not interested in discussing these options.

“That’s challenging because physicians won’t discuss it…There are different things to try without urgent things to cut. There can be openness to discuss things before surgery.” – Black woman participant

“Not everyone wants to be operated on, not everyone wants to go through surgeries. We need to make sure people talk about the alternatives.” – Black woman participant

However, one participant underwent a hysterectomy to stop her UF symptoms due to a decreased quality of life and wanted to continue her physical activities.

“I couldn’t wait for alternative medicine. I recently had a hysterectomy because I wanted to continue to race and didn’t want more children. Why should I suffer anymore?” – Black woman participant

All the participants agreed that discussions about their longitudinal goals with their provider were necessary to help them decide whether to undergo alternative treatments or surgical interventions.

“We need to ask what are your life goals? What do you plan to do? Are you willing to do this? Do you have time to make these changes? These are the important questions to ask when considering alternative treatment.” – Black woman participant

## Discussions

We conducted our CES to determine what should be included in the development of our interview guide for a national study on the menstruation and UF journey of NHB and HL. The following topics will be included in our interview guide: menstruation education, menarche experiences, menstrual symptoms, and UF symptomology, diagnosis, and treatment. Further probes will include if culture influenced any of the topics.

Many women talked about their early menstrual discussions, especially the lack of information and preparation for menarche. Mothers are the primary source for information on menstruation, yet some of our participants were not informed by their own mothers which was also reported in another study that included women across different cultural backgrounds [24, 25]. Our participants maintained feelings of discomfort in talking to family members and their physicians. Some women mentioned how parents assumed that the school system would provide information about menstruation, which has been an ongoing issue in the menstruation literature since 1980’s [26, 27]. Regardless of the source, menstrual education is needed to prevent negative menstrual experiences and bring awareness to abnormal menstrual symptoms [28, 29].

Many ethnoracially diverse women commented on the silence surrounding menstruation resulting in it being “taboo”. This is noted often in the literature [29–31]. Culture can perpetuate this “taboo” and negative attitude towards menstruation [32, 33]. The taboo around menstruation can be traced back to the 17th century with normal menstruation being perceived as a disorder [34]. Even to this day, menstruation is perceived as taboo that can be attributed to shame, embarrassment, and historical and religious context such as being associated as unclean and impure leading to restrictions [35, 36]. However, a way to overcome this “taboo” around menstruation is to begin normalizing and to publicly acknowledge the menstrual experience [37].

Participants believed that pain and heavy menstrual bleeding were “normal”, and as such did not report symptom onset to their healthcare providers, which was seen in work by Ghant and colleagues [38]. Heavy menstrual bleeding, referred to as abnormal uterine bleeding, refers to excessive menstrual blood loss which interferes with a women’s physical, social, emotional, and/or material quality of life [39]. Abnormal uterine bleeding is a symptom of many gynecological disorders and can serve as a warning sign to seek medical attention [40, 41]. Therefore, early education on “normal” menstruation and potential warning signs of UF and other disorders may facilitate earlier diagnosis and timely treatment for symptomatic patients.

One NHB participant mentioned being diagnosed with UF in the emergency room. This is not an uncommon experience, with NHB and HL being more likely to be diagnosed in the emergency room compared to NHW [42]. It is important to understand the root of this disparity in timely diagnosis. A possible cause may be how structural and individual racism impacts an NHB’s access to care and healthcare experience [43] but can also be traced to the “taboo” of menstruation leading to delays in menstruation consultations [31].

In our NHB CES, there was an interest in looking at alternative treatments for their UF, but participants met some difficulties with their physicians in implementing these treatments. Vannoy et al. echoes similar experiences in NHB when seeking surgical treatment for their UF [44]. At the time of diagnosis, NHB experience larger UF volumes and increased number of UF [45] resulting in NHB undergoing more drastic procedures like a hysterectomy at a higher rate [46, 47]. NHB are more likely to want to incorporate uterine-sparing procedures compared to NHW [48] but are sometimes not provided other nonsurgical treatment options. All the women agreed despite UF symptomology there needs to be a discussion between patients and providers to discuss which is the best treatment for their life goals. Incorporating the use of decision aids can help facilitate conversation to find the best course of treatment [49].

A strength of this project is the recruitment of ethnoracially diverse women and the opportunity to listen to their personal experiences around menstruation and uterine fibroids. Our goal was to determine areas of focus for a larger national study related to women’s health in ethnoracially diverse women in the United States. CES participants provided insight into the cultural influences, which have not been studied in the literature. Another strength was that the CES was done virtually which allowed us to recruit across the US without geographical limitations. Lastly, the group setting of the CES allowed participants to reflect on their own experiences based on others’ lived experiences, which may not have happened in a one-on-one interview.

One of the limitations is recall bias. Participants may not remember their exact experiences. However, this is a known limitation with qualitative research but is overcome by providing in-depth insights not captured by quantitative research. Another limitation is the incomplete demographics due to some participants not completing the CES post-survey. The women were also recruited via snowball sampling and may not be representative of the general population of ethnoracially diverse women, which limits generalizability. Another limitation is the distribution of quotes. Our UF quotes are exclusively from the NHB participants; this was due to our recruitment of NHB with UF as they experience a higher inequity. Both CES did include a cultural aspect around menstruation. However, the CES provided a foundation for future studies to continue exploring sociocultural influences on menstruation and UF along with potential areas for education about these topics.

## Conclusions

Participants mentioned the normalization of heavy bleeding due to the cultural “taboo” and stigma surrounding menstruation. Menstruation is a natural biological occurrence that is stigmatized leading to a lack of knowledge on “normal” menstrual symptoms. This leads to delays in UF diagnosis which results in more invasive procedures due to increased UF symptom burden. Further research needs to be done to determine how to disseminate proper menstrual education to women whether with their families, schools, or healthcare providers to present an accurate portrayal of menstrual symptoms. Understanding menstrual symptoms is the foundation to many gynecological diseases. Further education and policy supporting menstruating women is needed for awareness of abnormal menstrual symptoms, such as pain, to be able to seek gynecological care earlier. Tangible recommendations from participants included partnering with local Black and Latinx community organizations and hosting informational sessions.

Black women face a disparity in gynecological health including UF and endometrial cancer; early menstrual education is needed to decrease this inequity [45, 50]. Hispanic/Latinas have been understudied in the literature that further inclusion of this population to understand their gynecological health outcomes.

Community-engaged research is essential in conducting research with diverse populations. CES are a valuable way to include patient stakeholders in the development of a study. The results of the CES have provided further topics on menstruation and UF which have been incorporated in an interview guide that will use the socio-ecological model of health to delve deeper into influences in UF and menstruation.

## Data Availability

The datasets generated and/or analysed during the current study are not publicly available due to the participants not giving consent to having the information publically available but are available from the corresponding author on reasonable request.

## Abbreviations

CES: community engagement studios
HL: Hispanic/Latinas
NHB: Non-Hispanic Black/African American women
UF: uterine fibroids
US: United States
